# Comparison of postoperative plasma D‐dimer levels between patients undergoing laparoscopic resection and conventional open resection for colorectal cancer

**DOI:** 10.1111/ases.12796

**Published:** 2020-03-31

**Authors:** Yawara Kubota, Takashi Okuyama, Haruka Oi, Emiko Takeshita, Takashi Mitsui, Takuji Noro, Shinichi Sameshima, Tamaki Noie, Masatoshi Oya

**Affiliations:** ^1^ Department of Surgery, Saitama Medical Center Dokkyo Medical University Koshigaya Japan

**Keywords:** colorectal cancer, D‐dimer, laparoscopic surgery

## Abstract

**Introduction:**

D‐dimer is widely used in clinical pretests for venous thromboembolism exclusion, and its elevation suggests the presence of thrombus. The extent of hypercoagulability after colorectal surgery has not been systematically compared between patients who have undergone laparoscopic surgery and open surgery. The present study measured D‐dimer levels sequentially in patients undergoing colorectal surgery and compared the extent of hypercoagulability between laparoscopic surgery and open surgery.

**Methods:**

A prospective cohort study involving 169 patients who underwent resection of colorectal cancer at Saitama Medical Center, Dokkyo Medical University, was conducted between January 2013 and September 2014. To measure D‐dimer level, peripheral blood was obtained on postoperative day (POD) 1, POD4, and POD7. Enoxaparin sodium was administered twice daily as the routine prophylactic anticoagulant therapy on POD2 to 7.

**Results:**

D‐dimer levels on POD1, POD4, and POD7 were significantly higher after open surgery than after laparoscopic surgery. Older age, pathologically advanced stage cancer, greater intraoperative blood loss and higher preoperative D‐dimer levels were significantly associated with higher D‐dimer levels on POD1, POD4, and POD7. Patients who completed the course of postoperative enoxaparin injections had significantly lower D‐dimer levels on POD7 than those who did not receive postoperative enoxaparin injections. Multiple regression analyses of postoperative D‐dimer level showed that laparoscopic surgery was a significant and independent factor affecting D‐dimer level on POD4 and POD7.

**Conclusion:**

This study showed that postoperative D‐dimer levels were lower after laparoscopic surgery than after open surgery. The limited invasiveness of laparoscopic surgery may be beneficial to reduce the risk of postoperative deep vein thrombosis.

## 1 INTRODUCTION

Colorectal cancer is associated with hypercoagulability,^1^, ^2^ and colorectal surgery is known to carry an increased risk of deep vein thrombosis (DVT) in the postoperative period.^3^, ^4^ Because pulmonary embolism (PE) after DVT is a life‐threatening morbidity after major surgery, routine application of anticoagulant therapy is recommended.^5^

Compression ultrasonography and CT pulmonary angiography are currently considered the most reliable methods for diagnosing DVT and PE.^6^ However, performing such studies for all patients undergoing colorectal surgery is impractical. A more practical approach is examining D‐dimer level, a molecular marker for the presence of thrombus and secondary fibrinolysis. Increased concentrations suggest hypercoagulability, making plasma D‐dimer level suitable for clinical use to identify patients at high risk for DVT and PE in various situations.^6^

Although laparoscopic surgery is considered to be less invasive than conventional open surgery, the extent of hypercoagulability after colorectal resection has not been systemically compared between laparoscopic surgery and open surgery. The present study measured D‐dimer level sequentially in patients undergoing colorectal surgery and compared the extent of hypercoagulability between laparoscopic surgery and open surgery.

## 2 MATERIALS AND METHODS

The study included 169 patients (103 men, 66 women) who had undergone elective resection of colorectal cancer in the Department of Surgery at Saitama Medical Center, Dokkyo Medical University, between January 2013 and September 2014.

The median age of patients was 68.1 years (range, 36‐92 years). Of the 40 patients with rectal cancer, 30 underwent a sphincter‐preserving operation including Hartmann operation, and 10 underwent abdominoperineal resection. Laparoscopic resection was carried out in 115 patients, and conventional open surgery was performed in 54 patients. Patients who received an intravenous drip infusion of heparin preoperatively or underwent conversion from laparoscopic to conventional open surgery, synchronous gastrectomy, or synchronous resection of hepatic metastasis were excluded from the present investigation. Anti‐platelet medication was administered to 10 patients preoperatively and temporarily stopped at least 7 days before operation. On postoperative day (POD) 2 to POD7, 2000 IU of enoxaparin sodium was subcutaneously injected twice daily as the routine prophylactic anticoagulant therapy. This routine postoperative enoxaparin injection was completed in 137 patients; it was not performed at all in 27 patients for various reasons (eg, surgeon's preference) and terminated before POD7 in 5 patients because of clinical findings suggesting postoperative hemorrhagic complications. Clinically symptomatic DVT occurred postoperatively in only two patients in the present study.

To measure D‐dimer level, peripheral blood was obtained from the cubital vein in the morning on the day of operation and subsequently on POD1, POD4, and POD7. Plasma D‐dimer levels (LSI Medience Corporation, Tokyo, Japan) were determined in the routine manner in the clinical laboratory of the hospital. Clinical and pathological factors such as duration of operation, intraoperative blood loss, postoperative morbidity, and tumor stage were collected from hospital records. Pathological tumor stage was determined by using the criteria proposed by the Japanese Society for Colon and Rectum Cancer. Stages 0 and I were classified as “early stage,” and stages II, III, and IV were classified as “advanced stage.”

This study was approved by the institutional review board of Saitama Medical Center, Dokkyo Medical University. All patients gave written informed consent to the perioperative sequential measurement of D‐dimer level and the analysis of factors affecting preoperative D‐dimer level. 

### 2.1 Statistics analyses

Although the measured D‐dimer levels were not normally distributed, the distribution was almost normal after logarithmic transformation. Paired preoperative and postoperative plasma D‐dimer levels were compared by using the non‐parametric Wilcoxon signed‐rank correlation test. The correlation between two numerical variables was examined with Spearman's rank correlation coefficient. Multivariate analysis of factors affecting D‐dimer level was performed using multiple regression analysis in which the logarithmically transformed value of the D‐dimer level was the dependent variable. To test for independence, significant or close‐to‐significant (*P* < .2) factors identified on univariate analysis, such as age at operation, pathological stage, surgical approach (laparoscopic vs open), intraoperative bleeding, postoperative venous thromboembolism, and preoperative D‐dimer value, were entered into the multiple regression analysis by using a stepwise variable selection procedure. All statistical analyses were performed with the Dr. SPSS software package (SPSS Japan, Tokyo, Japan). *P* < .05 was considered to indicate a significant difference or correlation.

## 3 RESULTS

### 3.1 Comparison of clinical factors and pathological findings 

Patients undergoing open surgery were older than those undergoing laparoscopic surgery. In patients undergoing open surgery, pathological stages were more advanced than in those undergoing laparoscopic surgery. Other clinical factors did not differ significantly between the two surgical groups. The duration of operation was significantly longer and intraoperative blood loss was significantly less in patients who underwent laparoscopic surgery than in those who underwent open surgery. Preoperative anticoagulant therapy was marginally more often performed in patients undergoing open surgery than in those undergoing laparoscopic surgery. Postoperative anticoagulant therapy, postoperative incidence of surgical‐site infection, and postoperative incidence of venous thromboembolism did not differ significantly between the two groups (Table 1).

**Table 1:** Comparisons of backgrounds between patients after laparoscopic surgery and open surgery

	Laparoscopic (n = 115)	Open (n = 54)	*P* ‐value
Age at operation^a^ (y)	69 (60–73)	71 (66‐77)	.012
Gender (n)			
Male	71	32	
Female	44	22	.758
Tumor site (n)			
Colon/rectosigmoid	84	45	
Rectum	31	9	.142
Pathological stage (n)			
0‐I	43	10	
II‐IV	72	44	.014
Duration of operation^a^	218 (180‐282)	175 (126‐250)	<.001
Intraoperative bleeding^a^	30 (10‐70)	200 (60‐450)	<.001
Preoperative anticoagulant therapy (n)			
Not performed	111	48	
Ceased	4	6	.05
Postoperative anticoagulant therapy 9n)			
Not performed	15	12	
Incomplete	3	2	
Completed	97	40	.277
Postoperative SSI (n)			
Absent	105	47	
Present	10	7	.39
Postoperative VTE (n)			
Absent	114	53	
Present	1	1	.538

Abbreviations: SSI, surgical‐site infection; VTE, venous thromboembolism.

a Values show medians (interquartile ranges).

### 3.2 Comparisons of preoperative and postoperative D‐dimer levels

Preoperative D‐dimer levels were significantly higher in patients who underwent open surgery than in those who underwent laparoscopic surgery. Postoperative D‐dimer levels were significantly increased in both groups. D‐dimer levels on POD1, POD4, and POD7 were significantly higher after open surgery than after laparoscopic surgery (Table 2).

**Table 2:** Perioperative changes in D‐dimer levels in patients after laparoscopic surgery and open surgery

	Laparoscopic (n = 115)	Open (n = 54)	*P* ‐value
Preoperative	0.49 (0.27‐0.85)	1.26 (0.65‐2.35)	<.001
POD1	2.73 (1.74‐5.79)	4.19 (2.43‐7.01)	.005
POD4	2.53 (1.85‐3.99)	5.16 (3.25‐7.67)	<.001
POD7	2.81 (1.65‐3.74)	5.99 (3.47‐10.07)	<.001

*Note*: Values show medians (interquartile ranges).

Abbreviations: POD, postoperative day.

### 3.3 Factors correlated with postoperative D‐dimer levels

On POD1, POD4, and POD7, D‐dimer levels were significantly higher in elderly patients. Pathologically advanced stage cancer was significantly associated with higher D‐dimer levels on POD4 and POD7. A larger amount of intraoperative blood loss was significantly associated with higher D‐dimer levels on POD1, POD4, and POD7. Higher preoperative D‐dimer levels were significantly associated with higher postoperative D‐dimer levels on POD1, POD4, and POD7. Patients who completed the course of postoperative enoxaparin injections had significantly lower D‐dimer levels on POD7 than those who did not receive postoperative enoxaparin injections (Table 3).

**Table 3:** Correlations and comparisons of postoperative D‐dimer levels according to clinical and pathological factors

		POD1		POD4		POD7	
	n		*P*‐value		*P*‐value		*P*‐value
Age at operation	169	*R* = 0.261^a^	.001	*R* = 0.214^a^	.005	*R* = 0.199^a^	.009
Gender							
Male	103	3.05 (1.87‐6.35)	.935	3.37 (2.09‐5.86)	.437	3.43 (2.16‐5.94)	.652
Female	66	3.03 (1.91‐6.36)		3.07 (2.02‐5.57)		3.28 (1.95‐5.53)	
Tumor site							
Colon/rectosigmoid	129	3.32 (1.97‐6.48)	.115	3.14 (2.09–5.86)	.807	3.41 (2.19‐6.07)	.56
Rectum	40	2.61 (1.76‐5.09)		3.30 (2.03‐5.42)		3.43 (1.65‐5.57)	
Pathological stage							
0‐I	53	2.43 (1.51‐4.93)	.061	2.77 (1.87‐5.16)	.048	2.84 (1.53‐4.05)	.012
II‐IV	116	3.31 (2.16‐6.48)		3.35 (2.19‐6.31)		3.56 (2.39‐6.25)	
Duration of operation	169	*R* = 0.025^a^	.745	*R* = 0.088^a^	.255	*R* = 0.073^a^	.345
Intraoperative bleeding	169	*R* = 0.307^a^	<.001	*R* = 0.485^a^	<.001	*R* = 0.515^a^	<.001
Preoperative anticoagulant therapy							
Not performed	159	3.01 (1.85‐6.13)	.216	3.20 (2.07‐5.86)	.522	3.41 (1.98‐5.69)	.374
Ceased	10	4.72 (2.63‐5.92)		3.42 (2.63–5.92)		3.77 (2.50‐7.38)	
Postoperative anticoagulant therapy							
Not performed	27	6.71 (2.00‐12.85)		3.74 (2.69‐8.99)		5.41 (3.28‐15.8)	
Incomplete	5	4.85 (1.84‐5.37)	.106	3.75 (2.08‐4.90)	0.058	3.55 (1.72‐6.29)	.002
Completed	137	2.99 (1.86‐5.76)		3.09 (2.05‐5.45)		3.18 (1.87‐5.03)	
Postoperative SSI							
Absent	152	3.12 (1.86‐6.30)	.948	3.20 (2.06‐5.90)	0.983	3.34 (1.96‐65.91)	.255
Present	17	2.69 (2.10‐6.51)		3.20 (2.36‐4.41)		3.71 (3.21‐5.21)	
Postoperative VTE							
Absent	167	3.03 (1.87‐6.31)	.203	3.16 (2.08‐5.79)	0.088	3.42 (2.12‐5.63)	.311
Present	2	6.89 (5.72‐8.05)		12.4 (5.45‐18.64)		15.24 (3.18‐27.30)	
Preoperative DD level	169	*R* = 0.454^a^	<.001	*R* = 0.411^a^	<.001	*R* = 0.475^a^	<.001

*Note:* Unless otherwise indicated, values show medians (interquartile ranges).

Abbreviations: DD, D‐dimer; POD, postoperative day; SSI, surgical‐site infection; VTE, venous thromboembolism.

a Sperman's rank correlation.

### 3.4 Multiple regression analysis of factors correlated with postoperative D‐dimer levels

Table 4 shows the results of multiple regression analysis of the factors correlated with D‐dimer levels on POD1, POD4, and POD7. On POD1, preoperative D‐dimer level was the only factor correlated with D‐dimer level. On POD4, in addition to a lower preoperative D‐dimer level, laparoscopic surgery was associated with a lower D‐dimer level. On POD7, a lower preoperative D‐dimer level, laparoscopic surgery, and postoperative anticoagulant therapy were associated with a lower D‐dimer level.

**Table 4:** Results of multiple regression analyses on logarithmic values of postoperative D‐dimer levels

	Log (DD on POD1)		Log (DD on POD4)		Log (DD on POD7)	
	SRC	*P‐*value	SRC	*P‐*value	SRC	*P‐*value
Preoperative D‐dimer level	.391	<.001	0.274	<.001	.261	<.001
Laparoscopic vs open			0.291	<.001	.299	.001
Postoperative anticoagulant therapy					−.162	.017

Abbreviations: DD, D‐dimer; POD, postoperative day; SRC, standardized regression coefficient.

## 4 DISCUSSION

The incidence of DVT in the perioperative period of abdominal surgery has been reported to be 25% in a Western population and 23.7% in a Japanese population.^7^, ^8^ In the Japanese guideline for pulmonary thromboembolism and DVT, colorectal surgery for patients ≥40 years old is classified as a high‐risk procedure.^9^ Enoxaparin is therefore commonly administered in the postoperative period of colorectal surgery.

Compression ultrasonography and enhanced CT are established methods for the detection of DVT. Such imaging studies are recommended in patients who are likely to have DVT.^10^ Although the risk of postoperative DVT is regarded as high in most patients who undergo colorectal surgery, sequential ultrasonography of the lower extremities for all patients undergoing colorectal surgery is impractical. Further stratification of the risk of DVT after colorectal surgery is therefore considered likely to prove useful.

D‐dimer is a degradative product of fibrin by secondary fibrinolysis, and its elevation suggests the presence of thrombus. Although the value of D‐dimer level for the exclusion of DVT has been controversial, both standard and age‐adjusted cut‐off values show high sensitivity and a high negative predictive value.^11^

Postoperative elevation of the plasma D‐dimer level has been reported after cholecystectomy and hernia repair, even on POD28.^12^ In the present study, the plasma D‐dimer level was elevated until at least POD7 in most patients who had undergone colorectal surgery, but symptomatic DVT occurred in two patients. However, D‐dimer levels on POD1, POD4, and POD7 were significantly lower in patients after laparoscopic surgery than after open surgery. On multivariate analysis, surgical approach was a significant and independent factor affecting D‐dimer levels on POD4 and POD7.

Schietroma et al compared D‐dimer levels between patients after laparoscopic cholecystectomy and open cholecystectomy.^13^ Significantly higher D‐dimer levels were found in patients after open cholecystectomy than after laparoscopic cholecystectomy at 1 and 24 hours postoperatively. Using ultrasonography, Milic et al reported a higher incidence of DVT after open cholecystectomy than after laparoscopic cholecystectomy, although postoperative D‐dimer levels did not show any significant differences.^14^ Nguyen et al also reported that DVT was less frequent after laparoscopic surgery than after open surgery for appendicitis, cholelithiasis, cholecystitis, esophageal reflux, and morbid obesity.^15^ With regard to colectomy, Wilson et al reported a lower incidence of DVT after laparoscopic surgery than after open surgery.^16^ In the present study, only two patients—one from each group—presented with symptomatic DVT. However, lower postoperative D‐dimer levels after laparoscopic surgery than after open surgery may suggest a lower risk of DVT after laparoscopic colorectal cancer resection.

Among the other factors significantly correlated with postoperative D‐dimer level, preoperative D‐dimer level and postoperative anticoagulant therapy were significantly correlated with postoperative D‐dimer levels according to multiple regression analysis.

The strong correlation between preoperative and postoperative D‐dimer levels suggests that patients with preoperative hypercoagulability are prone to postoperative hypercoagulability. Stender et al reported that the cumulative incidence of DVT during the first year postoperatively was higher in patients with a preoperatively positive D‐dimer than in those with a preoperatively negative D‐dimer.^17^ Careful attention to postoperative DVT is therefore needed in patients with high D‐dimer levels before operation.

A correlation between histological stage and preoperative D‐dimer level was previously reported in patients with colorectal cancer.^18^ Preoperative D‐dimer is also reported to correlate with long‐term oncological outcomes after colorectal cancer resection.^19^, ^20^, ^21^, ^22^ On univariate analysis in the present study, tumor stage correlated with postoperative D‐dimer level.

In the present study, patients who received anticoagulant therapy, mainly enoxaparin, had lower postoperative D‐dimer levels than those who did not. This result supports the benefits of postoperative enoxaparin for preventing postoperative DVT.^5^, ^22^, ^23^ However, the risk of DVT reportedly increases 1 year after surgery.^17^ In the present study, routine measurement of plasma D‐dimer level was not possible because of restrictions imposed by the public medical insurance system in Japan.

A key limitation in the present study was that none of the patients underwent compression ultrasonography during the perioperative period. In addition, only two patients developed symptomatic DVT. Therefore, it is impossible to conclude that laparoscopic surgery is better than open surgery with regard to reducing the risk of DVT after colorectal resection. Another limitation is selection bias with regard to selecting laparoscopic or open surgery; this was not randomized in the present study. In addition, because this study was not conducted on consecutive patients, it might have caused further selection bias. Additional investigations are needed to determine the postoperative D‐dimer level that indicates the risk of postoperative DVT.

In conclusion, this study showed that postoperative D‐dimer levels were lower after laparoscopic surgery than after open surgery. The limited invasiveness of laparoscopic surgery may be beneficial to reduce the risk of postoperative DVT.

## DISCLOSURE

The authors have no conflicts of interest to declare.

## AUTHOR CONTRIBUTIONS

Y.K., T.O., and M.O. designed this study, the surgical procedures, and the data analysis. All authors participated in the surgical procedures. All authors read and approved the final manuscript.
